# Colorectal cancer incidences in Lynch syndrome: a comparison of results from the prospective lynch syndrome database and the international mismatch repair consortium

**DOI:** 10.1186/s13053-022-00241-1

**Published:** 2022-10-01

**Authors:** Pål Møller, Toni Seppälä, James G. Dowty, Saskia Haupt, Mev Dominguez-Valentin, Lone Sunde, Inge Bernstein, Christoph Engel, Stefan Aretz, Maartje Nielsen, Gabriel Capella, Dafydd Gareth Evans, John Burn, Elke Holinski-Feder, Lucio Bertario, Bernardo Bonanni, Annika Lindblom, Zohar Levi, Finlay Macrae, Ingrid Winship, John-Paul Plazzer, Rolf Sijmons, Luigi Laghi, Adriana Della Valle, Karl Heinimann, Elizabeth Half, Francisco Lopez-Koestner, Karin Alvarez-Valenzuela, Rodney J. Scott, Lior Katz, Ido Laish, Elez Vainer, Carlos Alberto Vaccaro, Dirce Maria Carraro, Nathan Gluck, Naim Abu-Freha, Aine Stakelum, Rory Kennelly, Des Winter, Benedito Mauro Rossi, Marc Greenblatt, Mabel Bohorquez, Harsh Sheth, Maria Grazia Tibiletti, Leonardo S. Lino-Silva, Karoline Horisberger, Carmen Portenkirchner, Ivana Nascimento, Norma Teresa Rossi, Leandro Apolinário da Silva, Huw Thomas, Attila Zaránd, Jukka-Pekka Mecklin, Kirsi Pylvänäinen, Laura Renkonen-Sinisalo, Anna Lepisto, Päivi Peltomäki, Christina Therkildsen, Lars Joachim Lindberg, Ole Thorlacius-Ussing, Magnus von Knebel Doeberitz, Markus Loeffler, Nils Rahner, Verena Steinke-Lange, Wolff Schmiegel, Deepak Vangala, Claudia Perne, Robert Hüneburg, Aída Falcón de Vargas, Andrew Latchford, Anne-Marie Gerdes, Ann-Sofie Backman, Carmen Guillén-Ponce, Carrie Snyder, Charlotte K. Lautrup, David Amor, Edenir Palmero, Elena Stoffel, Floor Duijkers, Michael J. Hall, Heather Hampel, Heinric Williams, Henrik Okkels, Jan Lubiński, Jeanette Reece, Joanne Ngeow, Jose G. Guillem, Julie Arnold, Karin Wadt, Kevin Monahan, Leigha Senter, Lene J. Rasmussen, Liselotte P. van Hest, Luigi Ricciardiello, Maija R. J. Kohonen-Corish, Marjolijn J. L. Ligtenberg, Melissa Southey, Melyssa Aronson, Mohd N. Zahary, N. Jewel Samadder, Nicola Poplawski, Nicoline Hoogerbrugge, Patrick J. Morrison, Paul James, Grant Lee, Rakefet Chen-Shtoyerman, Ravindran Ankathil, Rish Pai, Robyn Ward, Susan Parry, Tadeusz Dębniak, Thomas John, Thomas van Overeem Hansen, Trinidad Caldés, Tatsuro Yamaguchi, Verónica Barca-Tierno, Pilar Garre, Giulia Martina Cavestro, Jürgen Weitz, Silke Redler, Reinhard Büttner, Vincent Heuveline, John L. Hopper, Aung Ko Win, Noralane Lindor, Steven Gallinger, Loïc Le Marchand, Polly A. Newcomb, Jane Figueiredo, Daniel D. Buchanan, Stephen N. Thibodeau, Sanne W. ten Broeke, Eivind Hovig, Sigve Nakken, Marta Pineda, Nuria Dueñas, Joan Brunet, Kate Green, Fiona Lalloo, Katie Newton, Emma J. Crosbie, Miriam Mints, Douglas Tjandra, Florencia Neffa, Patricia Esperon, Revital Kariv, Guy Rosner, Walter Hernán Pavicic, Pablo Kalfayan, Giovana Tardin Torrezan, Thiago Bassaneze, Claudia Martin, Gabriela Moslein, Aysel Ahadova, Matthias Kloor, Julian R. Sampson, Mark A. Jenkins, The European Hereditary Tumour Group (EHTG) and the International Mismatch Repair Consortium (IMRC)

**Affiliations:** 1 Department of Tumor Biology, Institute of Cancer Research, The Norwegian Radium Hospital, 0379 Oslo, Norway; 2 Department of Gastrointestinal Surgery, Helsinki University Central Hospital, University of Helsinki, Helsinki, Finland; 3 Applied Tumour Genomics Research Program, University of Helsinki, Helsinki, Finland; 4 Faculty of Medicine and Health Technology, Tampere University and Tays Cancer Center, Tampere University Hospital, Tampere, Finland; 5 Centre of Epidemiology and Biostatistics, Melbourne School of Population and Global Health, The University of Melbourne, Melbourne, Victoria 3010 Australia; 6 Engineering Mathematics and Computing Lab (EMCL), Interdisciplinary Center for Scientific Computing (IWR), Heidelberg University, Heidelberg, Germany; 7 Data Mining and Uncertainty Quantification (DMQ), Heidelberg Institute for Theoretical Studies (HITS), Heidelberg, Germany; 8 Department of Clinical Genetics, Aalborg University Hospital, 9000 Aalborg, Denmark; 9 Department of Biomedicine, Aarhus University, DK-8000 Aarhus, Denmark; 10 Department of Surgical Gastroenterology, Aalborg University Hospital, Aalborg University, 9100 Aalborg, Denmark; 11 Institute of Clinical Medicine, Aalborg University Hospital, Aalborg University, 9100 Aalborg, Denmark; 12 Institute for Medical Informatics, Statistics and Epidemiology, University of Leipzig, 04107 Leipzig, Germany; 13 Institute of Human Genetics, National Center for Hereditary Tumor Syndromes, Medical Faculty, University of Bonn, 53127 Bonn, Germany; 14 Department of Clinical Genetics, Leids Universitair Medisch Centrum, 2300RC, Leiden, The Netherlands; 15 Hereditary Cancer Program, Institut Català d’Oncologia-IDIBELL, L; Hospitalet de Llobregat, 08908 Barcelona, Spain; 16 Division of Evolution and Genomic Sciences, Manchester Centre for Genomic Medicine, University of Manchester, Manchester University NHS Foundation Trust, Manchester, M13 9WL UK; 17 Translational & Clinical Research Institute, Faculty of Medical Sciences, Newcastle University, Newcastle upon Tyne, NE1 3BZ UK; 18 Campus Innenstadt, Medizinische Klinik und Poliklinik IV, Klinikum der Universität München, 80336 Munich, Germany; 19 MGZ – Center of Medical Genetics, 80335 Munich, Germany; 20 Division of Cancer Prevention and Genetics, IEO, European Institute of Oncology, IRCCS, 20141 Milan, Italy; 21 Department of Molecular Medicine and Surgery, Karolinska Institutet, 171 76 Stockholm, Sweden; 22 Department Rabin Medical Center, Service High Risk GI Cancer Gastroenterology, Petach Tikva, Israel; 23 Colorectal Medicine and Genetics, The Royal Melbourne Hospital, Melbourne, Australia; 24 Department of Medicine, Melbourne University, Melbourne, Australia; 25 The Royal Melbourne Hospital, Melbourne, Australia; 26 Department of Genetics, University of Groningen, University Medical Center Groningen, Groningen, The Netherlands; 27 Department of Medicine and Surgery, Laboratory of Molecular Gastroenterology, IRCCS Humanitas Research Hospital, University of Parma, Parma, Italy; 28 Hospital Fuerzas Armadas, Grupo Colaborativo Uruguayo, Investigación de Afecciones Oncológicas Hereditarias (GCU), Montevideo, Uruguay; 29 Medical Genetics, Institute for Medical Genetics and Pathology, University Hospital Basel, Basel, Switzerland; 30 Gastrointestinal Cancer Prevention Unit, Gastroenterology Department, Rambam Health Care Campus, Haifa, Israel; 31 Programa Cáncer Heredo Familiar Clínica Universidad de los Ande, Santiago, Chile; 32 University of Newcastle and the Hunter Medical Research Institute, Callaghan, Australia; 33 Department of Gastroenterology, Hadassah Medical Center, Faculty of Medicine, Hebrew University of Jerusalem, Jerusalem, Israel; 34 The Department of Gastroenterology, High Risk and GI Cancer Prevention Clinic, Gastro-Oncology Unit, Sheba Medical Center, Ramat Gan, Israel; 35 Hereditary Cancer Program (PROCANHE), Hospital Italiano de Buenos Aires, Ciudad Autónoma de Buenos Aires, Buenos Aires, Argentina; 36 Genomic and Molecular Biology Group, A.C.Camargo Cancer Center, Sao Paulo, Brazil; 37 Department of Gastroenterology, Tel-Aviv Sourasky Medical Center and Sackler Faculty of Medicine, Tel-Aviv University, Tel-Aviv, Israel; 38 The Institute of Gastroenterology and Hepatology, Soroka University Medical Center, Ben-Gurion University of the Negev, Beer Sheva, Israel; 39 St Vincent’s University Hospital, Elm Park, Dublin 4, Ireland; 40 Hospital Sirio Libanes, Sao Paulo, Brazil; 41 University of Vermont, Larner College of Medicine, Burlington, VT 05405 USA; 42 University of Tolima, Tolima, Colombia; 43 Foundation for Research in Genetics and Endocrinology, FRIGE House, Jodhpur Village Road, Satellite Ahmedabad, Ahmedabad, 380015 India; 44 Ospedale di Circolo ASST Settelaghi, Centro di Ricerca Tumori Eredo-Familiari, Università dell’Insubria, Varese, Italy; 45 Instituto Nacional de Cancerologia, Mexico, DF Mexico; 46 Department of Abdominal and Transplantation Surgery, Universitätsspital Zürich, Rämistrasse 100, CH-8091 Zürich, Switzerland; 47 Laboratório de Imonologia, ICS/UFBA, Núcleo de Oncologia da Bahia/Oncoclinicas, Salvador, Brazil; 48 Hospital Privado Universitario de Córdoba, Cordoba, Argentina; 49 Hospital Universitario Oswaldo Cruz, Universidade de Pernambuco, Hospital de Câncer de Pernambuco, IPON - Instituto de Pesquisas Oncológicas do Nordeste, Salvador, Brazil; 50 Department of Surgery and Cancer, St Mark’s Hospital, Imperial College London, London, UK; 51 Department of Transplantation and Surgery, Semmelweis University Budapest, Budapest, Hungary; 52 Faculty of Sport and Health Sciences, University of Jyväskylä, Jyväskylä, Finland; 53 Department of Surgery, Central Finland Health Care District, Jyväskylä, Finland; 54 Department of Education and Science, Central Finland Health Care District, Jyväskylä, Finland; 55 Department of Medical and Clinical Genetics, University of Helsinki, Helsinki, Finland; 56 The Danish HNPCC Register, Gastro Unit, Copenhagen University Hospital – Amager and Hvidovre, Copenhagen, Denmark; 57 Department of Applied Tumour Biology, Institute of Pathology, University Hospital Heidelberg, Heidelberg, Germany; 58 Clinical Cooperation Unit Applied Tumour Biology, German Cancer Research Center (DKFZ), Heidelberg, Germany; 59 Institute for Medical Informatics, Statistics and Epidemiology, University of Leipzig, Leipzig, Germany; 60 Institute of Human Genetics, University Clinic Düsseldorf, Heinrich-Heine-University, Düsseldorf, Germany; 61 Department of Medicine, Knappschaftskrankenhaus, Ruhr-University Bochum, Bochum, Germany; 62 Department of Internal Medicine, University Hospital Bonn, Bonn, Germany; 63 Genetics Unit, Hospital Vargas de Caracas, Caracas, Venezuela; 64 Escuela de Medicina Jose Maria Vargas, Universidad, Central de Venezuela, UCV, Caracas, Venezuela; 65 St Mark’s Hospital, London, UK; 66 Department of Clinical Genetics, Rigshospitalet, Copenhagen University Hospital, DK-2100 Copenhagen, Denmark; 67 Department of Medicine Solna, Unit of Internal medicine, Karolinska Institutet, Stockholm, Sweden; 68 Medical Oncology Department, Hospital Universitario Ramón y Cajal, IRYCIS, Madrid, Spain; 69 Hereditary Cancer Center, Department of Preventive Medicine, Creighton University, Omaha, NE 68178 USA; 70 Murdoch Children’s Research Institute and University of Melbourne Department of Paediatrics, Royal Children’s Hospital, Parkville, VIC 3052 Australia; 71 Department of Genetics, Brazilian National Cancer Institute, Rio de Janeiro, Brazil; 72 Molecular Oncology Research Center, Barretos Cancer Hospital, Barretos, São Paulo, Brazil; 73 Department of Internal Medicine, University of Michigan, Ann Arbor, MI USA; 74 Department of Clinical Genetics, Amsterdam University Medical Centers, University of Amsterdam, Meibergdreef 9, 1105 AZ Amsterdam, The Netherlands; 75 Department of Clinical Genetics, Fox Chase Cancer Center, Philadelphia, PA USA; 76 Division of Human Genetics, The Ohio State University Comprehensive Cancer Center, Columbus, OH 43210 USA; 77 Department of Urology, Geisinger Medical Center, Danville, PA 17822 USA; 78 Department of Molecular Diagnostics, Aalborg University, Aalborg, Denmark; 79 Department of Genetics and Pathology, International Hereditary Cancer Center, Pomeranian Medical University in Szczecin, Szczecin, Poland; 80 Lee Kong Chian School of Medicine, Nanyang Technological University Singapore and Cancer Genetics Service National Cancer Centre Singapore, Singapore, Singapore; 81 Gastrointestinal Surgery, University of North Carolina, Chapel Hill, NC USA; 82 New Zealand Familial Gastrointestinal Cancer Service, Auckland, New Zealand; 83 St Mark’s Hospital & Imperial College, London, UK; 84 Ohio State University Comprehensive Cancer Center, Columbus, OH 43210 USA; 85 Department of Cellular and Molecular Medicine, Center for Healthy Aging, University of Copenhagen, Copenhagen, Denmark; 86 Department of Clinical Genetics, Amsterdam UMC, Vrije Universiteit Amsterdam, Amsterdam, Netherlands; 87 IRCCS AOU di Bologna, and Department of Medical and Surgical Sciences - University of Bologna, Bologna, Italy; 88 Woolcock Institute of Medical Research, Glebe, Sydney, NSW 2037 Australia; 89 Department of Human Genetics and Department of Pathology, Radboud University Medical Center, Nijmegen, the Netherlands; 90 Monash Health Translation Precinct, Monash University, Clayton South, VIC 3169 Australia; 91 Zane Cohen Centre, Sinai Health System, Toronto, Ontario Canada; 92 Faculty of Health Sciences, University Sultan Zainal Abidin, Kuala Terengganu, Terengganu Malaysia; 93 Division of Gastroenterology and Hepatology, Mayo Clinic, Phoenix, AZ 85054 USA; 94 Adelaide Medical School, University of Adelaide, Adelaide, SA 5000 Australia; 95 Adult Genetics Unit, Royal Adelaide Hospital, Adelaide, SA 5000 Australia; 96 Department of Human Genetics, Radboud University Medical Center, Nijmegen, The Netherlands; 97 Regional Medical Genetics Centre, Belfast HSC Trust, City Hospital Campus, Queen’s University Belfast, Belfast, Northern Ireland UK; 98 Peter MacCallum Department of Oncology, The University of Melbourne, Parkville, VIC 3010 Australia; 99 Genomics Platform Group, Centre for Cancer Research, University of Melbourne, Parkville, VIC Australia; 100 The Biology Department, Ariel University, Ariel and the Oncogenetic Clinic, The Clinical Genetics Institute, Kaplan Medical Center, Rehovot, Israel; 101 Human Genome Centre, School of Medical Sciences, Universiti Sains Malaysia, Kubang Kerian, Kelantan Malaysia; 102 Department of Laboratory Medicine and Pathology, Mayo Clinic Arizona, Scottsdale, AZ 85259 USA; 103 Faculty of Medicine and Health, University of Sydney, Sydney, NSW 2006 Australia; 104 Department of Genetics and Pathology, Pomeranian Medical University in Szczecin, Szczecin, Poland; 105 Department of Medical Oncology, Peter MacCallum Cancer Centre, Melbourne, Victoria Australia; 106 Molecular Oncology Laboratory, Hospital Clínico San Carlos, IdISSC, Madrid, Spain; 107 Department of Clinical Genetics, Tokyo Metropolitan Cancer and Infectious Diseases Center Komagome Hospital, Tokyo, Japan; 108 Department of Genetics, Hospital Universitario Ramón y Cajal, IRYCIS, Madrid, Spain; 109 Gastroenterology and Gastrointestinal Endoscopy Unit, Vita-Salute San Raffaele University, San Raffaele Scientific Institute, Milan, Italy; 110 Technische Universität Dresden, Dresden, Germany; 111 Department of Pathology, University Hospital of Cologne, Cologne, Germany; 112 Department of Health Science Research, Mayo Clinic Arizona, Phoenix, USA; 113 Lunenfeld Tanenbaum Research Institute, Mount Sinai Hospital, University of Toronto, Toronto, Canada; 114 University of Hawaii Cancer Center, Honolulu, USA; 115 Public Health Sciences Division, Fred Hutchinson Cancer Research Center, Seattle, WA 98109-1024 USA; 116 Colorectal Oncogenomics Group, Department of Clinical Pathology, The University of Melbourne, Parkville, Victoria Australia; 117 University of Melbourne Centre for Cancer Research, Victorian Comprehensive Cancer Centre, Parkville, Victoria Australia; 118 Genomic Medicine and Family Cancer Clinic, Royal Melbourne Hospital, Parkville, Victoria Australia; 119 Department of Laboratory Medicine and Pathology, Mayo Clinic, Rochester, MN 55905 USA; 120 Leids Universitair Medisch Centrum, Leiden, Netherlands; 121 Department of Informatics, Centre for Bioinformatics, University of Oslo, Oslo, Norway; 122 Centre for Cancer Cell Reprogramming (CanCell), Institute of Clinical Medicine, Faculty of Medicine, University of Oslo, Oslo, Norway; 123 Department of Surgery, Central Manchester University Hospitals NHS Foundation Trust and University of Manchester, Manchester, UK; 124 Gynaecological Oncology Research Group, Manchester University NHS Foundation Trust, Manchester, UK; 125 Division of Cancer Sciences, University of Manchester, Manchester, UK; 126 Division of Obstetrics and Gyneacology, Department of Women’s and Children’s Health, Karolinska Institutet, Karolinska University Hospital, Solna, Stockholm, Sweden; 127 Instituto de Medicina Traslacional e Ingeniería Biomédica (IMTIB), Hospital Italiano de Buenos Aires-IUHI-CONICET, Ciudad Autónoma de Buenos Aires, Buenos Aires, Argentina; 128 Surgical Center for Hereditary Tumors, Ev. Bethesda Khs Duisburg, University Witten-Herdecke, Herdecke, Germany; 129 Division of Cancer and Genetics, Institute of Medical Genetics, Cardiff University School of Medicine, Heath Park, Cardiff, CF14 4XN UK

**Keywords:** Lynch Syndrome, Epidemiology, Prevention, Penetrance, Colorectal cancer, Segregation analysis, Prospective, Incidence, Over-diagnosis, Colonoscopy

## Abstract

**Objective:**

To compare colorectal cancer (CRC) incidences in carriers of pathogenic variants of the MMR genes in the PLSD and IMRC cohorts, of which only the former included mandatory colonoscopy surveillance for all participants.

**Methods:**

CRC incidences were calculated in an intervention group comprising a cohort of confirmed carriers of pathogenic or likely pathogenic variants in mismatch repair genes (*path_MMR)* followed prospectively by the Prospective Lynch Syndrome Database (PLSD). All had colonoscopy surveillance, with polypectomy when polyps were identified. Comparison was made with a retrospective cohort reported by the International Mismatch Repair Consortium (IMRC). This comprised confirmed and inferred *path_MMR* carriers who were first- or second-degree relatives of Lynch syndrome probands.

**Results:**

In the PLSD, 8,153 subjects had follow-up colonoscopy surveillance for a total of 67,604 years and 578 carriers had CRC diagnosed. Average cumulative incidences of CRC in *path_MLH1* carriers at 70 years of age were 52% in males and 41% in females; for *path_MSH2* 50% and 39%; for *path_MSH6* 13% and 17% and for *path_PMS2* 11% and 8%. In contrast, in the IMRC cohort, corresponding cumulative incidences were 40% and 27%; 34% and 23%; 16% and 8% and 7% and 6%. Comparing just the European carriers in the two series gave similar findings. Numbers in the PLSD series did not allow comparisons of carriers from other continents separately. Cumulative incidences at 25 years were < 1% in all retrospective groups.

**Conclusions:**

Prospectively observed CRC incidences (PLSD) in *path_MLH1* and *path_MSH2* carriers undergoing colonoscopy surveillance and polypectomy were higher than in the retrospective (IMRC) series, and were not reduced in *path_MSH6* carriers. These findings were the opposite to those expected. CRC point incidence before 50 years of age was reduced in *path_PMS2* carriers subjected to colonoscopy, but not significantly so.

**Supplementary Information:**

The online version contains supplementary material available at 10.1186/s13053-022-00241-1.

## Background

In 1995, a study in Finland found that colonoscopy with polypectomy conducted every three to five years was associated with a reduced CRC incidence in Lynch syndrome (LS) when compared to LS patients who did not have colonoscopy [1]. It was stated that ‘*The recommended surveillance protocol for HNPCC is based on the hypothesis that the adenoma-carcinoma sequence, which is generally accepted in sporadic colorectal cancer, is also applicable in HNPCC’* [2] and relatives of LS cases were thereafter widely subjected to surveillance with colonoscopy every three years. However, continued occurrence of CRC was noted despite this surveillance and, in response, the recommended interval between colonoscopies was reduced [3, 4]. Despite this change, continuing occurrence of CRC was still observed and in some centres the interval between colonoscopies was therefore reduced further, to one year [5] or even less. Surprisingly, since the initial Finnish report in 1995 that did not control for lead-time bias and was not randomized, there has been no confirmatory study to show that colonoscopy surveillance with polypectomy significantly reduces CRC incidence in LS.

The earliest published reports of LS families [6] suggest that in previous generations, most individuals who developed a first cancer died from that cancer. By contrast, more recently, LS patients diagnosed with a non-colorectal cancer at a young age usually survive and often develop CRC later in life [7]. The extent to which this time-trend (survivor bias) has influenced the outcomes of interventions that aim to prevent occurrence of CRC and improve its prognosis through early detection and treatment, is not known.

No single institution nor country had the resources needed to resolve these issues, leading the European Hereditary Tumour Group in 2012 (www.ehtg.org, that at the time was known as the Mallorca group) to invite pooling of international results of prospective follow-up of LS families in a single shared database, the Prospective Lynch Syndrome Database (PLSD). The goal of PLSD was to describe cancer incidences in all organs in carriers of *path_MMR* variants who were undergoing follow-up according to the internationally advocated clinical guidelines and to stratify these by age, gene and gender. Once sufficient numbers of carriers and follow-up years were collated, the intention was to use the information obtained to assess whether the results were compatible with current assumptions about carcinogenesis and the expected effects of interventions in LS. This paper reports the results of one such assessment. Because neither randomized trials of colonoscopy versus no-colonoscopy, nor open trials with a non-intervention control arm are likely to be undertaken in LS, a separate goal of PLSD is to produce the information needed to inform development of alternative randomized trials, for example of different surveillance intervals, in the future.

Around the same time as the PLSD was developed, an initiative aiming to compile data on as many LS families as possible for a retrospective segregation analysis was established by the International Mismatch Repair Consortium (IMRC) (https://www.sphinx.org.au/imrc ). Its primary aim was to determine the cumulative CRC incidences in *path_MMR* carriers by retrospective analysis including family members in former generations who were not subject to the same degree of CRC preventive surveillance with colonoscopy as contemporary patients followed in PLSD [5].

There is limited information on survival following CRC detected during surveillance with colonoscopy in *path_MMR* carriers, other than reports from the PLSD [8] and the recent IMRC report did not include data on survival [5].

Here, we compare prospective CRC incidences in an updated version of PLSD that includes 8,153 *path_MMR* carriers aged from 25 to 70 years and subjected to regular follow-up with colonoscopy for a total of 67,604 years with retrospective CRC incidences calculated from *path_MMR* carriers from 5,255 families collected by the IMRC.

## Methods

The PLSD compiles observed cancers in *path_MMR* carriers from the first prospectively planned and performed colonoscopy. It considers all cancers that occur before or at the same age as the first colonoscopy as prior or prevalent cancers, and from that point onwards it counts new primary cancers as events. Data collection was made from age 25 years at earliest, and cumulative incidence of CRC at age 25 years was set to zero. When CRC was counted as the event, all carriers who already had CRC prior to or at inclusion in the study were excluded, and observation time was right-censored at the first event, last observation or death, whichever came first. These methods have been discussed in detail in a separate report [9]. Lead-time bias was controlled by colonoscopy at inclusion and only scoring CRC after inclusion as an event, but since there is no pre-determined time to right-censoring and no obligatory colonoscopy at right-censoring is required, length-time bias may occur. Although the median observation time was less than 10 years, as it is longer in some patients, time-trend bias may occur. Except for the effects of ascertainment bias, which will affect any study, the annual incidences of CRC reported by PLSD are not subject to any assumptions underlying the calculations: they reflect observed events divided by observation years in each age group. Observation years and events (i.e. CRCs) in each five-year age group were calculated by using MySQL80 ©. Incidence rates (AIR) were calculated in five-year cohorts starting at age 25 as number of events (in this paper CRCs) divided by number of observation years in each age cohort. The corresponding incidence risk (IR) was approximated by IR = AIR × 5 years. Cumulative incidence risk was set to zero at age 25. In previous PLSD reports cumulative incidence, denoted Q, was computed starting at age 25 using the formula Q(age) = Q(age − 1) + [1 − Q(age − 1)] × AIR(age) and the 95% confidence intervals (CIs) were calculated using the Lagrange multiplier test. In this report, we calculated the cumulative incidence risks and the 95% CIs based on Nelson-Aalen estimates with an underlying Poisson distribution as detailed in the supplementary note. As expected, the cumulative incidences calculated by the former and present methods were close to identical, while the Poisson distribution gave slightly different CIs (comparison not shown). There were insufficient numbers to calculate results separately for continents other than Europe.

The IMRC used a segregation analysis to study a retrospective family cohort of first- and second-degree relatives, including 31,944 first-degree relatives and 47,865 second-degree relatives of *path_MMR* probands in 5,585 families. Most probands were *path_MLH1* or *path_MSH2* carriers, and the methods were discussed previously. Pedigree data of *path_MMR* families was sought from clinicians and researchers worldwide between July 11, 2014 and December 31, 2018 [5]. Observation time for all individuals included their lifetime risk of first colorectal cancer from birth to their age at death or last known age at latest update to the cohort in 2018. In summary, standard methods correcting for ascertainment bias and then calculating cumulative incidences of CRC by age, gene and gender were applied. The results were reported by continent. To allow for direct comparison with the PLSD, the results of IMRC segregation analyses for the current study were not right-censored at polypectomy (as had been done in the previously published IMRC report [5]) and consequently the results presented here may differ slightly from those previously published. The overall (global) averages were calculated as a weighted mean of the results from the different continents that were previously reported.

## Results

Cumulative incidences at ages 30, 40, 50, 60 and 70 years for male and female carriers by MMR gene are detailed in Table 1. For the PLSD series, numbers of carriers included and follow-up years by country are given in the supplementary Table 1.

**Table 1:** Percent cumulative incidences (with 95% confidence intervals in parentheses) of CRC at ages 30, 40, 50, 60 and 70 years by gender and genetic variant for both series for European *path_MMR* carriers separately and for all carriers irrespective of residence, and overall cumulative incidence at 25 years in the IMRC series

		% cumulative incidences CRC (95% confidence intervals)						
Sex	Gene	Continent	25 years	30 years	40 years	50 years	60 years	70 years
Male	*MLH1*	Europe IMRC		0.9 (0.4-1.8)	2.6 (1.2-5.2)	8.1 (5.0-14)	21 (15-29)	36 (27-48)
		Europe PLSD		2.7 (1.0-7.0)	14.2 (10.2-19.6)	30.3 (25.0-36.6)	44.3 (37.9-51.2)	49.2 (42.0-56.8)
		All IMRC	0.7 (0.5-1.1)	1.5 (1.1-2.2)	5.8 (4.3-7.8)	15 (12-19)	27 (23-33)	40 (34-47)
		All PLSD		3.5 (1.6-7.6)	14.8 (10.9-20.0)	32.1 (27.0-38.0)	45.1 (39.2-51.4)	51.9 (45.2-58.9)
	*MSH2*	Europe IMRC		0.7 (0.4-1.5)	2.2 (1.1-4.2)	6.7 (4.1-11)	16 (12-22)	28 (21-38)
		Europe PLSD		2.7 (0.9-8.1)	8.9 (5.3-14.6)	19.1 (13.9-25.9)	34.1 (26.8-42.8)	46.4 (37.1-56.7)
		All IMRC	0.6 (0.4-0.8)	1.2 (0.9-1.8)	4.8 (3.6-6.5)	13 (10-16)	23 (19-28)	34 (28-40)
		All PLSD		3.7 (1.6-8.8)	10.4 (6.8-15.9)	21.3 (16.3-27.4)	37.3 (30.8-44.6)	49.6 (41.5-58.4)
	*MSH6*	Europe IMRC		0.4 (0.1-1.3)	1.2 (0.4-3.8)	3.6 (1.4-9.7)	8.1 (4.1-17)	14 (7.8-24)
		Europe PLSD		3.1 (0.4-20.1)	4.6 (1.1-18.5)	5.9 (1.8-18.6)	11.5 (5.5-23.5)	11.5 (5.5-23.5)
		All IMRC	0.2 (0.1-0.4)	0.4 (0.2-0.9)	1.6 (0.9-3.2)	4.8 (3.0-8.7)	9.9 (7.0-16)	16 (12-24)
		All PLSD		2.8	6.0	7.1	11.8	13.4
				(0.4-18.3)	(1.9-18.1)	(2.6-18.7)	(5.9-22.9)	(7.0-24.9)
	*PMS2*	Europe IMRC		0.1 (0.1-0.1)	0.3 (0.3-0.3)	1.0 (0.9-1.1)	3.5 (2.8-4.1)	7.6 (5.7-9.7)
		Europe PLSD		0 (-)	0 (-)	0 (-)	12.1 (3.2-40.3)	12.1 (3.2-40.3)
		All IMRC	0.0 (0.0-0.0)	0.1 (0.1-0.1)	0.3 (0.3-0.3)	1.1 (1.0-1.1)	3.3 (3.0-3.6)	7.1 (6.3-8.1)
		All PLSD		0 (-)	0 (-)	0 (-)	10.7 (2.8-36.5)	10.7 (2.8-36.5)
Female	*MLH1*	Europe IMRC		0.4 (0.2-0.9)	1.3 (0.6-2.9)	4.7 (2.6-8.2)	12 (8.4-18)	22 (15-32)
		Europe PLSD		0 (-)	8.5 (5.7-12.7)	17.8 (13.8-22.8)	29.9 (24.7-35.9)	41.0 (34.7-48.0)
		All IMRC	0.4 (0.3-0.7)	0.8 (0.5-1.2)	3.1 (2.2-4.5)	8.6 (6.6-12)	17 (14-21)	27 (22-33)
		All PLSD		0 (-)	9.3 (6.5-13.2)	18.2 (14.5-22.9)	29.9 (25.1-35.4)	41.3 (35.4-47.8)
	*MSH2*	Europe IMRC		0.4 (0.2-1.0)	1.5 (0.7-3.3)	4.7 (2.6-8.9)	10 (6.7-17)	17 (11-27)
		Europe PLSD		2.3 (0.7-6.9)	7.2 (4.1-12.4)	15.3 (10.8-21.4)	23.3 (17.8-30.1)	38.6 (31.4-46.9)
		All IMRC	0.4 (0.3-0.7)	0.8 (0.6-1.3)	3.3 (2.3-5.0)	8.7 (6.5-12)	15 (12-20)	23 (19-29)
		All PLSD		1.8 (0.6-5.4)	7.1 (4.3-11.6)	15.4 (11.4-20.6)	23.4 (18.6-29.1)	38.7 (32.5-45.6)
	*MSH6*	Europe IMRC		0.1 (0.0-0.2)	0.2 (0.1-0.7)	0.8 (0.3-2.3)	2.5 (1.3-5.5)	5.6 (2.8-11)
		Europe PLSD		0 (-)	0 (-)	3.1 (1.0-9.5)	8.2 (4.4-15.3)	14.5 (8.7-23.5)
		All IMRC	0.0 (0.0-0.1)	0.1 (0.0-0.2)	0.3 (0.1-1.0)	1.0 (0.6-3.0)	3.4 (2.4-6.4)	8.1 (5.5-13)
		All PLSD		0 (-)	1.2 (0.2-8.2)	4.0 (1.5-10.4)	8.4 (4.6-15.2)	16.8 (10.8-25.5)
	*PMS2*	Europe IMRC		0.1 (0.1-0.1)	0.2 (0.2-0.2)	0.9 (0.8-0.9)	2.6 (2.2-3.0)	5.3 (4.2-6.6)
		Europe PLSD		0 (-)	0 (-)	0 (-)	0 (-)	3.4 (0.5-21.7)
		All IMRC	0.0 (0.0-0.0)	0.1 (0.1-0.1)	0.3 (0.3-0.3)	1.0 (1.0-1.0)	2.7 (2.5-2.9)	5.6 (5.0-6.2)
		All PLSD		0 (-)	0 (-)	0 (-)	0 (-)	7.9 (1.9-29.3)

### Prospective PLSD series under colonoscopy surveillance

In total 8,153 carriers were subject to follow-up with colonoscopy for 67,604 years with mean follow-up time of 8.3 years, including 6,266 carriers followed-up for 53,559 years with mean follow-up time 8.5 years in Europe. Five-hundred and seventy-eight carriers had CRC diagnosed. Average cumulative incidences of CRC (with 95% confidence intervals in parentheses) in *path_MLH1*, *path_MSH2*, *path_MSH6* and *path_PMS2* carriers at 70 years of age were 52 (45-59)%/ 41 (35-48)%, 50 (42-58)%/ 39 (33-46)%, 13 (7-25)%/ 17 (11-26)% and 11 (3-37)%/ 8 (2-29)% in male/female carriers in the total cohort, respectively. For carriers followed-up in Europe, the corresponding cumulative incidences at 70 years were 49 (42-57)% / 41 (35-48)%, 46 (37-57)%/ 39 (31-47) %, 12 (6-24)%/ 15 (9-24)% and 12 (3-40) %/ 3 (1-22)%, respectively.

### Retrospective IMRC series

The corresponding average calculated cumulative incidences up to 70 years of age based on all carriers from the IMRC cohort were 40 (34-47)%/ 27 (22-33)%, 34 (28-40)%/ 23 (19-29)%, 16 (12-24)%/ 8 (6-13)% and 7 (6-8)%/ 6 (5-6)%, respectively. The cumulative incidences in families from Europe were 36 (27-48)%/ 22 (15-32)%, 28 (21-38)%/ 17 (11-27)%, 14 (8-24)%/ 6 (3-11)% and 8 (6-10)%/ 5 (4-7)%, respectively. Cumulative incidences at 25 years of ages were < 1.0% in all of the groups considered. Sixty percent of the carriers were right-censored after 1980.

**Fig. 1:**
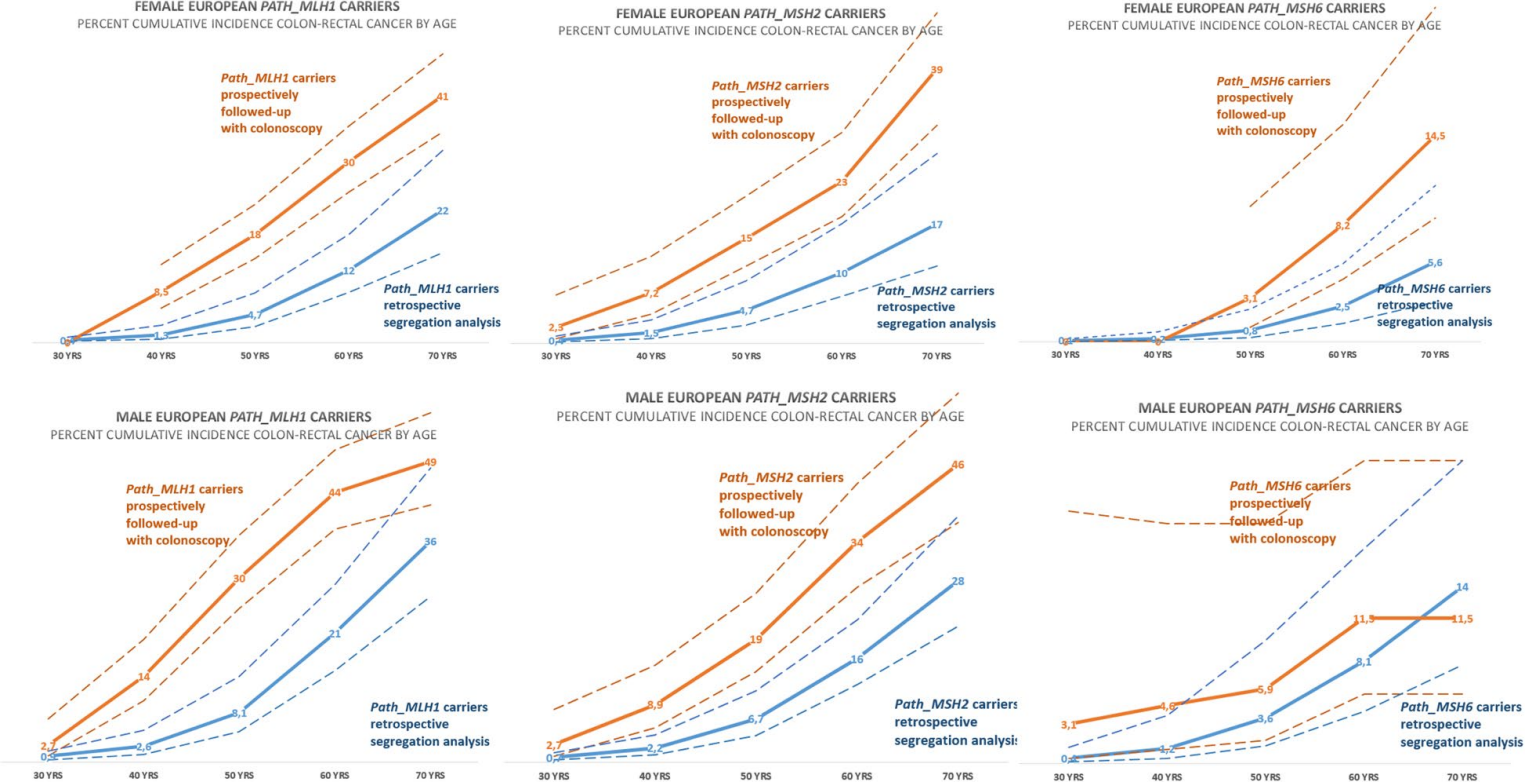
Cumulative incidences of CRC by genetic variant and gender in PLSD (the prospective series with colonoscopy) and IMRC (the retrospective series) in European *path_MLH1, path_MSH2* and *path_MSH6* carriers. Broken lines indicate 95% confidence intervals

As seen in Table 1 and Fig. 1, the cumulative incidences of CRC in *path_MLH1* and *path_MSH2* carriers of both genders were significantly higher in the prospective PLSD cohort in which all were subjected to regular colonoscopy surveillance than in the IMRC cohort (95% confidence intervals do not overlap). No significant differences were observed for *MSH6,* for which fewer patients and events were available in both cohorts (95% confidence intervals of the one series overlap the mean of the other). The point estimates for the mean for *path_PMS2* carriers below 50 years of age indicated a lower CRC incidence in the PLSD cohort when compared to the IMRC cohort, but this was not statistically significant.

## Discussion

Prospectively observed incidences of CRC in *path_MLH1*, *path_MSH2* and *path_MSH6* carriers of both genders in the PLSD cohort, in which all patients were subject to colonoscopy surveillance, were up to twice as high as in the retrospective IMRC series that included carriers who did not all receive regular surveillance colonoscopy.

Consideration of the methodologies used and the associated statistical concepts and confounders is indicated to explore the possibility that the results we obtained might reflect methodological biases, particularly as they were the opposite of what was expected. The PLSD methods have been described previously [9] and the IMRC results were produced using commonly accepted methods, as previously described [5]. Assuming the PLSD mean observation time (8.3 years) to be applicable to IMRC cases right-censored after 1980 (when surveillance with colonoscopy was introduced) as a maximum estimate of the fraction of IMRC cases subjected to colonoscopy, at least 85% of the IMRC observation years would have been completed without colonoscopy. The main finding of the current study would not, anyway, be confounded if a fraction of cases in the IMRC cohort underwent colonoscopy. We recognize that some individuals will have been included in the PLSD as well as the IMRC cohort, but we cannot identify them and their inclusion in both cohorts will not have contributed to the differences in observed CRC incidences. It is possible that CRC was under-reported in the pedigrees obtained by clinical teams, the details of which constituted the primary data source for the IMRC analysis. By contrast, under-reporting of CRC is unlikely in the PLSD cohort whose subjects were under regular surveillance at the contributing centres. It is unlikely that the lower CRC incidences estimated in the IMRC cohort are due to differing frequencies of lower penetrance *path_MMR* variants or modifying genes in former generations because there have been no known major fluctuations (bottlenecks) in population sizes or structures over the last three generations to cause such changes. As 60% of IMRC cases were right-censored after 1980, possible lower CRC incidence in previous generations (time-trend bias) can not explain the results. However, Lynch syndrome colorectal cancer incidence is associated with lower physical activity [10], and higher body mass index [11]. Temporal changes in such factors could explain some of the observed differences in colorectal cancer incidences between the two cohorts.

In a previous retrospective study, segregation analyses describing cumulative incidences of CRC in French *path_MLH1*, *path_MSH2* and *path_MSH6* carriers [12] were not restricted to first and second degree relatives. This helped to avoid simply returning the criteria used to identify families for genetic testing as the results of the study, and to minimize the effects of removing young affected carriers when considering a family to have a hypergeometric distribution of events. Observation time was right-censored at the diagnosis of any cancer in order to avoid survival bias when deaths from other cancers were caused by the same genetic variant. Additional family members were tested and demonstrated to carry the genetic variant in question in order to minimize the confounder of there being additional inherited causes of cancer within the family. Observation time was also right censored if the carrier was subjected to surveillance colonoscopy in order to avoid the confounder of colonoscopy modifying CRC incidence. The point estimates of cumulative CRC incidence at 70 years in that report for both genders combined were 41% for *path_MLH1* carriers, 48% for *path_MSH2* carriers and 12% for *path_MSH6* carriers, very close to the observed incidences in the PLSD series in the current report. The number of cases included was, however, limited and the confidence intervals correspondingly wide – a reason why the PLSD waited for the IMRC results to be available before making a comparison of its prospective data with results of retrospective segregation analyses. While the French report could suggest that the IMRC segregation analyses have underestimated CRC incidence, comparing the current PLSD results with the point estimates in the French series does not demonstrate any reduction in CRC incidence associated with colonoscopy surveillance in the PLSD cohort.

A European multicentre segregation analysis that estimated CRC incidence in *path_PMS2* carriers [13] demonstrated an increased incidence in carriers under 50 years of age, similar to the findings reported in the IMRC series. In a subsequent report [14] the same group confirmed that the apparent anticipation observed was a statistical artifact caused by birth cohorts. The PLSD design eliminates such artificial anticipation. The *path_PMS2* carriers in the PLSD cohort had lower incidence of CRC before 50 years of age than those reported by the IMRC, but not significantly so. That is, the assumption that colonoscopy reduces CRC incidence may be true for younger adult *path_PMS2* carriers. If this finding is confirmed, the recently revised clinical guidelines for *path_PMS2* carriers [15] that advocate postponing surveillance compared to other groups with LS would need to be reconsidered. Observations in larger numbers of *path_PMS2* carriers are needed to clarify this.

A recent overview of current knowledge on carcinogenetic mechanisms in LS CRCs [16] reported that in addition to the traditional adenoma-carcinoma pathway [2], other carcinogenetic mechanisms also need to be considered. Five hypotheses were described. including 1) adenomas that are overlooked during colonoscopy, 2) fast progression of adenomas to carcinomas [2], 3) CRCs developing without a macroscopically visible adenoma phase 4) over-diagnosis / disappearing cancers [17] and 5) colonoscopy inducing cancer in *path_MMR* carriers via damage of the colonic epithelium. Hypothesis 1 and 2 cannot explain the results described in this paper as we found higher rates of CRC incidence in those receiving colonoscopy. Although hypothesis 5 is consistent with our results, we have no method to evaluate this. We are left with hypotheses 3 and 4, that CRC may develop directly from MMR deficient crypts without a macroscopically visible precursor and that microsatellite instable crypts, or more advanced cancers, may be invaded by immunocompetent cells leading to their eradication. The latter underlies the principle of neoadjuvant checkpoint inhibitor therapy that has shown marked success in recent trials in MMR deficient CRCs [18, 19] and current studies exploring the feasibility of vaccines to prevent or cure LS cancers [20]. An adult *path_MLH1* or *path_MSH2* carrier is thought to have > 1000 microsatellite instable crypts in his/her colon [21–24]. It is known that apparently healthy *path_MMR* carriers have measurable immune responses against frameshift-induced neo-peptides, suggesting their immune systems can detect and potentially attack microsatellite instable crypts [25]. The probabilities for such crypts persisting, disappearing or developing into infiltrating cancers are not known. The biology of CRC in *path_PMS2* carriers may be different from carriers of the pathogenic variants of the other genes [26, 27].

Although the focus of this paper is on CRC incidence, we consider prevention of death due to CRC to be the ultimate goal of surveillance colonoscopy, and the good prognosis of CRC detected in *path_MMR* carriers who are subjected to colonoscopy every three years or more frequently has been described in previous PLSD reports [8]. This is a strong argument to continue surveillance of *path_MMR* carriers by colonoscopy. The current paper does not call this into question, but its findings do support a change in the message to be communicated to *path_MMR* carriers, namely that the purpose of surveillance colonoscopy is not to prevent CRC from occurring but to detect it early. The authors of the current study have previously examined the relationship between colonoscopy interval and CRC incidence, stage at diagnosis, and survival [18, 28–30] without finding evidence of lower CRC incidence, less advanced stage or better survival when the interval between colonoscopies is shortened to less than three years.

Lastly, the very low incidence of CRC before 25 years of age that was found in the IMRC cohort indicates that the PLSD methodology of setting CRC incidence to zero at 25 years of age was justified. Indeed, without extensive genetic testing, one cannot exclude the possibility that occurrence of CRC before 25 years of age may, in some cases, have been due to inclusion of unrecognized biallelic *path_MMR* carriers (i.e. individuals who were affected by constitutional mismatch repair deficiency syndrome) or the presence of pathogenic germline variants in other co-existing CRC predisposition genes [31].

The strength of the current study is that it compares the results of the two largest studies to date on CRC incidences in *path_MMR* carriers. One weakness is the lack of information on colonoscopy in the IMRC series: contributors to IMRC were asked to provide information on colnosocpy sceeening and polypectomy, however this was not provided for the vast majority of submitted individuals. Another weakness is lack of information on the degree to which follow up for cases included in the PLSD complied with recommendations. The effects of non-compliance would be to diminish the differences between the two series and could not explain the increased CRC incidence in the PLSD series compared to the IMRC series.

## Conclusions

We found a higher incidence of CRC in the carriers reported to PLSD, all of whom received colonoscopic surveillance. However, as the details of colonoscopies that will have been undertaken in some of the IMRC cohort are not available, we cannot quantify the magnitude of this effect. Although these findings could reflect differences in the fidelity of recording of CRC in the retrospective and prospective cohorts, the findings could also be explained by the occurrence of carcinogenetic mechanisms in LS CRC that override the preventive effect of colonoscopy [16].

## Supplementary Information

**Additional file 1.** 

## Data Availability

Not applicable.

## Abbreviations

LS: Lynch syndrome
CRC: colorectal cancer
*Path_MMR*: pathogenic or likely pathogenic variant of one of the *MLH1, MSH2, MSH6* or *PMS2* genes
*Path_MLH1*: pathogenic or likely pathogenic variant of one of the *MLH1* gene
*Path_MSH2*: pathogenic or likely pathogenic variant of one of the *MLH1* gene
*Path_MSH6*: pathogenic or likely pathogenic variant of one of the *MLH1* gene
*Path_PMS2*: pathogenic or likely pathogenic variant of one of the *MLH1* gene
PLSD: The Prospective Lynch Syndrome Database
IMRC: The International Mismatch Repair Consortium
