# Supplementary material for: Colorectal cancer incidences in Lynch syndrome: a comparison of results from the prospective lynch syndrome database and the international mismatch repair consortium
Source: Hered Cancer Clin Pract. 2022 Oct 1;20:36. doi: 10.1186/s13053-022-00241-1 (PMC9526951; doi:10.1186/s13053-022-00241-1)
Supplement: Supplementary file 1 — Additional file 1. [file 13053_2022_241_MOESM1_ESM.docx]

Supplementary Table 1

Number of *path_MMR* carriers, average follow-up years and mean age at inclusion by country

| **Country** | **Number carriers** | **Follow-up years** | **Mean follow-up years** | **Mean age at**  **inclusion** |
| --- | --- | --- | --- | --- |
| DENMARK | 1,644 | 15,871 | 9.7 | 42.8 |
| FINLAND | 1,035 | 13,555 | 13.1 | 41.1 |
| GERMANY | 976 | 6,633 | 6.8 | 43.9 |
| AUSTRALIA | 794 | 6,875 | 8.7 | 44.7 |
| SPAIN | 675 | 3,531 | 5.2 | 44.5 |
| UK | 564 | 3,727 | 6.6 | 43.8 |
| HOLLAND | 543 | 3,760 | 6.9 | 49.0 |
| USA | 361 | 2,337 | 6.5 | 49.6 |
| ITALY | 305 | 2,548 | 8.4 | 40.1 |
| NORWAY | 286 | 2,039 | 7.1 | 43.2 |
| ISRAEL | 277 | 1,538 | 5.6 | 43.4 |
| CANADA | 171 | 1,255 | 7.3 | 48.7 |
| SWEDEN | 148 | 1,375 | 9.3 | 43.1 |
| SWITZERLAND | 71 | 444 | 6.3 | 49.6 |
| URUGUAY | 68 | 502 | 7.4 | 43.1 |
| NEW_ZEALAND | 60 | 448 | 7.5 | 44.0 |
| BRAZIL | 55 | 459 | 8.3 | 45.5 |
| CHILE | 44 | 265 | 6.0 | 43.9 |
| ARGENTINA | 34 | 281 | 8.3 | 41.6 |
| IRELAND | 18 | 70 | 3.9 | 48.2 |
| COLOMBIA | 12 | 59 | 4.9 | 47.2 |
| INDIA | 8 | 11 | 1.4 | 43.9 |
| MEXICO | 3 | 15 | 5.0 | 28.3 |
| HUNGARY | 1 | 6 | 6.0 | 39.0 |
|  |  |  |  |  |
| **Sum** | **8153** | **67604** | **8.3** |  |

|  |  |  |  |  |
| --- | --- | --- | --- | --- |

# **Statistical methods for estimating cumulative incidence risks and corresponding confidence intervals based on a Poisson distribution**

Saskia Haupt and Vincent Heuveline

Engineering Mathematics and Computing Lab (EMCL), Interdisciplinary Center for Scientific Computing (IWR), Heidelberg University, Heidelberg, Germany, and Data Mining and Uncertainty Quantification (DMQ), Heidelberg Institute for Theoretical Studies (HITS), Heidelberg, Germany

This document summarizes the methods used in this paper to estimate cumulative incidence risks and the corresponding confidence intervals based on a Poisson distribution. It is mainly based on Chapter 2 in (Collett, 2014).

## Methods

The data consists of a number of subjects, each followed for a known time period, either until an event occurs or until they leave the study for other reasons. In other words, cancer is assumed to be a dichotomous variable (cancer occurs yes or no). The number of events, i.e., cancer cases, and time at risk are then aggregated per 5-year age interval starting from 25 to 75 years of age. This is mathematically described by a Poisson distribution.

### Incidence rates and cumulative incidence

We use the number of observed cancer cases $d$ and the number of observation years $y$ in the 5-year cohorts to compute the incidence rates $\mathrm{AIR}$ within the corresponding 5-year age intervals by $AIR= d / y$. The corresponding incidence risk $\mathrm{IR}$ which is typically used in survival analysis is approximated by $IR = AIR 5 yrs$.

To make use of the Poisson distribution, we use the Nelson-Aalen estimator of the cumulative hazard function in age interval $k$, which is given by

$\hat{H}\left( t_{k} \right)=\sum_{j=1}^{k} \mathrm{IR}\left( t_{j} \right)$,

to compute the quantity of interest, namely the Nelson-Aalen estimate for the distribution function of the random variable $T$ associated with the survival time. In our setting, the latter is called cumulative cancer incidence estimate given by

$\hat{F}\left( t_{k} \right)=1-\exp\left( -\hat{H}\left( t_{k} \right) \right)$.

### Standard error and point-wise confidence intervals

Uncertainties of the above point estimates are computed and presented as standard errors and confidence intervals.

With the rules for calculating variances and the Delta method, it holds for the variance of the Nelson-Aalen estimator of the cumulative hazard function

$\mathrm{Var}\hat{H}\left( t_{k} \right)=Var \sum_{j=1}^{k} \frac{5d_{j}}{y_{j}}=\sum_{j=1}^{k} \mathrm{Var}\frac{5d_{j}}{y_{j}}=\sum_{j=1}^{k} \frac{25d_{j}}{y_{j}^{2}}$,

where the summation is over all 5-year age intervals $j$ up to the age interval $k$ of interest.

A point-wise confidence interval can be obtained by assuming that the Nelson-Aalen estimate at a given point in time is a sample from a normal distribution. We use the logarithmic transformation which was shown empirically to perform well for this kind of data, in particular for small sample sizes (Bie, Borgan, & Liestøl, 1987). For each point estimate in age interval $k$, we first compute the 95% confidence interval for the cumulative hazard function

$\left[ \hat{H}_{\mathrm{lower}}\left( t_{k} \right),\hat{H}_{\mathrm{upper}}\left( t_{k} \right) \right]=\left[ \hat{H}\left( t_{k} \right)\exp\left( -z_{0.975}\frac{\sqrt{\mathrm{Var}\hat{H}\left( t_{k} \right)}}{\hat{H}\left( t_{k} \right)} \right),\hat{H}\left( t_{k} \right)\exp\left( z_{0.975}\frac{\sqrt{\mathrm{Var}\hat{H}\left( t_{k} \right)}}{\hat{H}\left( t_{k} \right)} \right) \right]$,

which is feasible for $\hat{H}\left( t_{k} \right)\neq0$ and thus,

$\left[ \hat{F}_{\mathrm{lower}}\left( t_{k} \right),\hat{F}_{\mathrm{upper}}\left( t_{k} \right) \right]=\left[ 1-\exp\left( -\hat{H}_{\mathrm{lower}}\left( t_{k} \right) \right),1-\exp\left( -\hat{H}_{\mathrm{upper}}\left( t_{k} \right) \right) \right]$.

In general, we could use the $1-\alpha/ 2$ percentile $z_{1-\alpha/2}$ of the standard normal distribution to obtain confidence interval estimates for a $1-\alpha$ confidence level.

# References

Bie, O., Borgan, Ø., & Liestøl, K. (1987). Confidence Intervals and Confidence Bands for the Cumulative Hazard Rate Function and Their Small Sample Properties. Scandinavian Journal of Statistics, Vol. 14, No. 3 (1987), pp. 221-233.

Collett, D. (2014). Modelling Survival Data in Medical Research. New York: Chapman and Hall/CRC.
